# Effectiveness of community-based interventions for older adults living alone: a systematic review and meta-analysis

**DOI:** 10.4178/epih.e2024013

**Published:** 2024-01-03

**Authors:** Inhye Kim, Hyunseo An, Sohyeon Yun, Hae Yean Park

**Affiliations:** 1 Department of Occupational Therapy, Graduate School, Yonsei University, Wonju, Korea; 2 Department of Occupational Therapy, College of Software and Digital Healthcare Convergence, Yonsei University, Wonju, Korea

**Keywords:** Aged, Loneliness, Meta-analysis, Health behavior, Community-based participatory research, Health policy

## Abstract

**OBJECTIVES:**

This study examined the effectiveness of community-based interventions designed for older adults living alone through a systematic review and meta-analysis.

**METHODS:**

The study incorporated 4 randomized controlled trials (RCTs) and 5 non-RCTs to evaluate various interventions. The methodological quality of these studies was assessed using the Downs and Black checklist, while Q-statistic and I-square tests were performed to examine statistical heterogeneity. Additionally, visual inspection of funnel plots and the trim-and-fill method were employed to investigate potential publication bias. Of the 2,729 identified studies, 9 met the criteria for inclusion in this review. Independent variables were categorized into 5 groups (physical activity, nutrition, social relationships, social participation, and combined intervention) to examine their effects. Dependent variables were similarly classified into 5 subgroups to identify the specific effects of the interventions.

**RESULTS:**

Interventions focusing on nutrition and combined approaches were the most effective, yielding effect sizes of 0.96 (95% confidence interval [CI], 0.66 to 1.25) and 0.43 (95% CI, 0.26 to 0.60), respectively. The interventions had the greatest impacts on the health behavior and mental health of the participants, with effect sizes of 0.98 (95% CI, 0.73 to 1.22) for health behavior and 0.67 (95% CI, 0.19 to 1.16) for mental health.

**CONCLUSIONS:**

This study suggests a direction for the development of community-based interventions tailored to the needs of older adults living alone. Additionally, it provides evidence to inform policy decisions concerning this demographic.

## INTRODUCTION

Over recent decades, the number of people living alone has risen, particularly among the older adult population [1]. Data from the U.S. Census Bureau America’s Families and Living Arrangements publication indicate that 28% of all United States households consist of 1 person [2]. Furthermore, 27% of United States adults aged 60 years and older live alone, and this proportion increases with age. For instance, approximately 44% of women aged 75 years and older live alone [3].

Previous studies have indicated that living alone may lead to mental health issues, including deficits in cognitive function, depression, social isolation, and impaired memory and coping skills [4-6]. The loss of spouses or friends and transitions to different living arrangements can further contribute to feelings of loneliness and depression [7-9]. Moreover, older adults who live alone face comparatively high risks of illness and safety concerns [4,10]. Physical incidents, such as falls, within community-dwelling populations are often accompanied by negative emotional states, such as loneliness or depression [11-13]. Another major concern is the prevalence of poverty among older adults living alone [14-16]. Data from the Organization for Economic Cooperation and Development in 2019 revealed that 13.5% of adults over the age of 65 years were experiencing poverty, a figure that rose to 14.1% by 2021 [17,18]. Such financial challenges can restrict access to adequate healthcare services and support.

To mitigate the aforementioned issues faced by older adults living alone, it is essential to implement supportive policies and community programs [19,20]. Concerning the former, several countries have enacted policies specifically targeting this demographic. For instance, Europe, Australia, and the United States have introduced co-housing policies designed to facilitate community living among older adults [21]. Nevertheless, these policies have primarily concentrated on offering financial assistance and reducing social isolation, resulting in a notable absence of policies aimed at health promotion and disease prevention [22-24].

Related research has indicated that interventions promoting a healthy lifestyle are more impactful than financial assistance in promoting successful aging among older adults who live alone [1]. Additionally, a separate review noted that interventions aimed at health promotion can improve the quality of life and health of solitary older adults [25]. The studied interventions included physical activities, occupational engagement, and dietary guidance. Healthcare professionals, including occupational therapists, delivered these interventions with the objective of enhancing the health of older adults living independently.

Although these approaches were effective, their success was mixed due to the lack of detailed classification of the intervention fields [25]. These fields, which include physical activities, occupational engagement, and nutritional advice, are closely associated with factors that contribute to a healthy lifestyle [26]. These factors can be categorized into 4 subgroups: physical activity, nutrition, social relationships, and social participation [27,28]. Physical activity and nutrition play key roles in preventing disease and disability [29-31], while active social relationships and diverse social participation positively impact mental health [32-34]. Although interventions overall have demonstrated a beneficial effect on healthy lifestyles, the specific impact attributable to each type of approach has not been reported. To better understand the effects of community-based interventions on healthy lifestyles, these interventions should be classified into subgroups based on lifestyle factors and analyzed to determine their respective effect sizes. In this study, lifestyle factors were segmented into physical activity, nutrition, social relationships, and social participation to examine their individual effects.

In the present study, we undertook a meta-analysis to assess the effectiveness of community-based lifestyle interventions aimed at older adults who live alone [35]. To determine the effect sizes of the interventions, the independent variables were organized into 5 groups based on lifestyle factors. Concurrently, the outcome measures were classified into 5 distinct subgroups.

## MATERIALS AND METHODS

This systematic review was conducted in accordance with the Preferred Reporting Items for Systematic Reviews and Meta-Analyses (PRISMA) guidelines [36].

### Search strategy

From January 30, 2023 to January 31, 2023, we performed a literature search of 3 databases: PubMed, Embase, and the American Congress of Rehabilitation Medicine (ACRM). The search terms employed were: “elderly (MeSH)” OR “old adult*” AND “living alone” OR “live alone” OR “lives alone” OR “lived alone” AND “intervention.” We applied a 10-year time restriction to the search results in each database and removed duplicates using EndNote 20 (Clarivate Analytics, London, UK). Subsequently, we screened the studies for relevance using their titles and abstracts, adhering to established inclusion and exclusion criteria.

#### Inclusion and exclusion criteria

The studies selected for review met the following inclusion criteria [37]: (1) research on community-based interventions targeting older adults who reside alone; (2) experimental designs that included comparisons of data before and after the implementation of community-based interventions; (3) study participants who were at least 60 years old and who lived alone; (4) the measurement of at least 1 quantitative outcome resulting from a community-based intervention; (5) articles written in the English language; (6) studies for which the full text was accessible; and (7) research published within the past 10 years. Studies were excluded based on the following criteria: (1) literature reviews, observational studies, and conference abstracts; (2) studies with insufficient data; and (3) research focusing on surgical or pharmacological interventions.

#### Data selection and data synthesis

After the search, identification, and removal of duplicate studies, the remaining articles were independently evaluated by 2 authors in accordance with the PRISMA 2020 guidelines, as well as the inclusion and exclusion criteria [36]. In instances of differing selections, the authors engaged in discussions to reach a consensus on inclusion. The selected studies underwent further assessment for eligibility and were analyzed based on the population, intervention, comparison, outcome, and study design strategy before being finalized.

#### Assessment of selection bias

The studies included in the analysis underwent quality assessment utilizing the Downs and Black checklist [38]. This checklist serves as a tool for evaluating methodological quality and selection bias in both randomized and non-randomized studies. It comprises 27 items designed to evaluate a study’s quality, external validity, internal validity with respect to bias, internal validity concerning confounding factors, and statistical power. The maximum achievable score on this checklist is 32, with higher scores indicating greater methodological quality.

#### Meta-analysis

This study included a meta-analysis, conducted to statistically evaluate the effects of community-based interventions on older adults living alone. All analyses were carried out using Comprehensive Meta-Analysis version 4 (Biostat, Englewood, NJ, USA).

#### Statistical heterogeneity

We assessed the statistical heterogeneity of the variables related to community-based lifestyle interventions. To do so, we conducted an I-square (I^2^) test for statistical heterogeneity and examined homogeneity using the Cochran Q-statistic [39]. If the p-value of the Q-statistic fell below 0.1 and the I^2^ value exceeded 50%, the effect size was deemed heterogeneous, and thus, a random-effects model was applied. If these criteria were not met, a fixed-effects model was utilized [40].

#### Categorizing the independent and dependent variables

The interventions and outcomes of the included studies were classified to determine the effects of community-based interventions on older adults. The types of interventions were divided into 5 groups: physical activity, nutrition, social relationships, social participation, and combined intervention. Similarly, the outcomes were divided into 5 categories: health behavior, mental health, perceived health, physical health, and social-emotional health.

#### Effect sizes

Means, standard deviations, sample sizes, and p-values were utilized to determine the overall effect size. Effect sizes were depicted through forest plots employing Z-values. Here, the Z-values were applied to assess the null hypothesis, which posited that the mean effect size was equal to zero. Specifically, should the alpha criterion fall below 0.05, the null hypothesis would be rejected, indicating that the mean effect size is not zero. The Cohen d was also used to compare effect sizes across intervention types and outcomes.

#### Publication bias

Publication bias refers to the tendency for studies demonstrating stronger effects to be published more frequently than those showing weaker effects. In this study, we assessed publication bias using funnel plots and the trim-and-fill method. Specifically, funnel plots were employed to examine the symmetry of the data and to detect any potential bias. The trim-and-fill method was utilized to estimate the number of studies contributing to publication bias, after which we compared the estimated mean effect sizes. If the difference in the estimated effect size was 0.1 or greater, we considered publication bias to be present.

### Ethics statement

This study employs a meta-analysis research methodology, utilizing data provided by previously published papers. As it does not involve human subjects, institutional review board approval is not required.

## RESULTS

### Study selection and risk of bias

**Figure 1.**
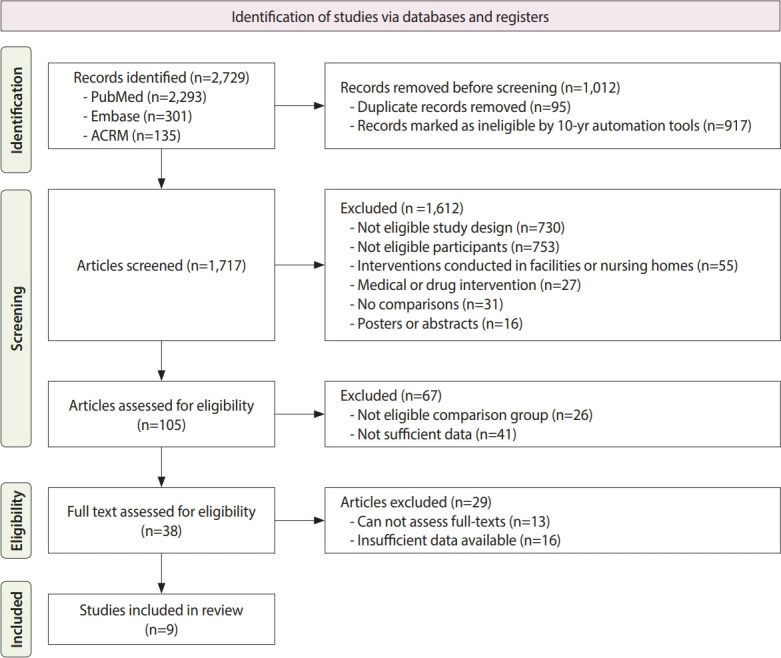
Preferred Reporting Items for Systematic Reviews and Meta-Analyses 2020 flow diagram for the systematic review. ACRM, American Congress of Rehabilitation Medicine.

A total of 2,729 studies were identified across the 3 databases (PubMed, Embase, and ACRM). Subsequently, 1,012 articles were removed as duplicated or because they were published prior to 2013. Of the remaining 1,717 documents, 1,612 were excluded after initial screening, and an additional 67 records were discarded. Following the assessment of their eligibility for this study, an additional 29 articles were excluded. The study selection process is detailed in Figure 1.

The remaining 9 studies were assessed regarding their risk of selection bias using the Downs and Black checklist [38]. The mean± standard deviation score on this checklist was 28.95± 1.76 out of 32, which falls within the first quartile. This score suggests that the included studies were of high quality. Of the studies assessed, 4 were randomized controlled trials (RCTs) [41-44], while the other 5 were non-RCTs [37,45-48]. Notably, most of these studies did not address randomization and blinding procedures, which are critical for supporting the quality of results by minimizing subjective bias. Nevertheless, as all studies achieved high scores on the aforementioned checklist, none were disqualified from inclusion.

Among the 9 studies reviewed, 4 took place in Korea, with 1 each in Finland, Spain, Turkey, Sweden, and Norway. These studies included a total of 546 older adults (65 years and older, without cognitive impairments) who were living alone and who received community-based interventions. More than 80% of the participants were women. Furthermore, only 1 study specifically included older women living alone, whereas multiple studies involved participants who were classified as frail, pre-frail, or having chronic pain [47] (Supplementary Material 1).

A total of 38 datasets were obtained from the 9 studies, which were then categorized into 5 lifestyle subgroups: physical activity, nutrition, social relationships, social participation, and combined intervention. This categorization was performed to examine the effect sizes produced by specific types of community-based interventions. Within these studies, 10 interventions were focused on physical activity, 5 on nutrition, 5 on social relationships, and 2 on social participation, while 16 involved a combination. Combined interventions were the most common among the community-based approaches, followed by physical activity and social relationship interventions. In terms of outcomes, the 9 studies reported on a variety of health aspects: 6 datasets pertained to health behavior, 5 to mental health, 4 to perceived health, 10 to physical health, and 13 to social-emotional health. Social-emotional and physical health outcomes were the most frequently measured, while health behavior, mental health, and perceived health outcomes were assessed to a lesser extent (Supplementary Material 1).

### Meta-analysis

#### Statistical heterogeneity

The I^2^ statistic was computed at 67.9%, suggesting that 67.9% of the variability in observed effects can be attributed to true variability in effect rather than to sampling error. The Q-value was computed at 115.27 with 37 degrees of freedom (p< 0.001). Consequently, this study employed a random-effects model to determine the effect sizes of the community-based interventions for older adults living alone (Tables 1 and 2).

#### Effect sizes of the community-based interventions

**Figure 2.**
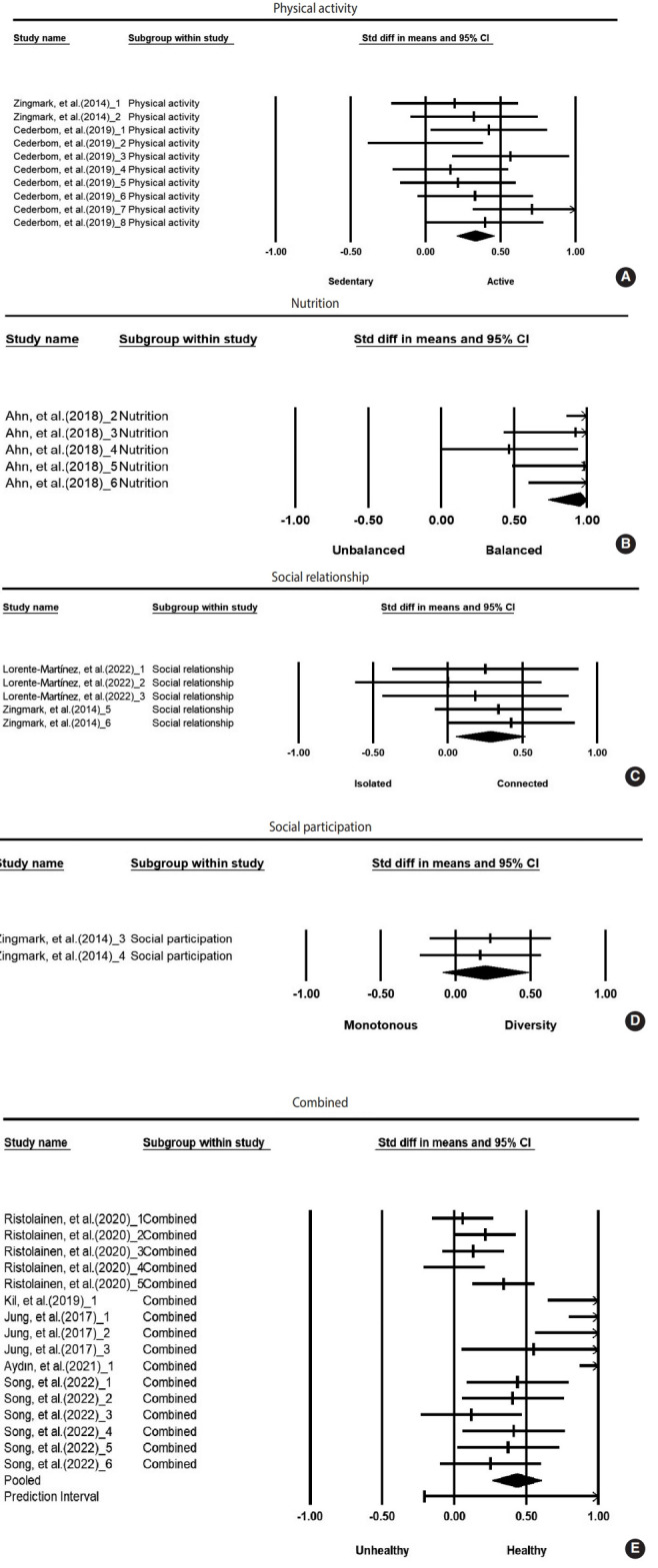
Forest plots for each type of intervention (A) physical activity, (B) nutrition, (C) social relationship, (D) social participation, and (E) combined. Std diff, standard difference; CI, confidence interval.

The interventions were categorized into 5 groups: physical activity, nutrition, social relationships, social participation, and combined intervention. The effect sizes for these subgroups were as follows: physical activity had an effect size of 0.33 (95% confidence interval [CI], 0.20 to 0.46); nutrition, 0.96 (95% CI, 0.67 to 1.25); social relationships, 0.29 (95% CI, 0.06 to 0.52); social participation, 0.20 (95% CI, -0.09 to 0.48); and combined intervention, 0.43 (95% CI, 0.26 to 0.60). The overall effect size for interventions was 0.38 (95% CI, 0.32 to 0.54). All intervention types demonstrated statistically significant effect sizes, with the exception of social participation (Table 1 and Figure 2).

The effect sizes of the outcome-dependent variables, categorized by type of outcome, were as follows: health behavior exhibited an effect size of 0.98 (95% CI, 0.73 to 1.22); mental health, 0.68 (95% CI, 0.19 to 1.16); perceived health, 0.48 (95% CI, -0.03 to 0.98); physical health, 0.32 (95% CI, 0.20 to 0.44); and social-emotional health, 0.24 (95% CI, 0.13 to 0.35). The overall effect size for outcomes was 0.43 (95% CI, 0.32 to 0.54). All outcome measures displayed statistically significant effect sizes, except for perceived health (Table 2).

### Publication bias

**Figure 3.**
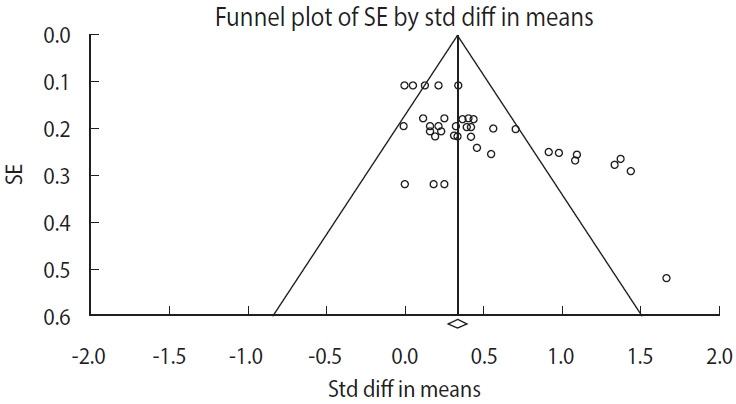
Funnel plot illustrating the relationship between standard error (SE) and standard difference (std diff) in means.

The funnel plot depicted in Figure 3 suggests a bias toward the left side, whereas 7 outcomes demonstrated a bias toward the right side, within a 95% CI. The trim-and-fill test revealed no discrepancy between the observed and adjusted mean values on the left side. Furthermore, the difference between the observed and adjusted mean values on the right side was negligible, at -0.00. Consequently, the findings indicate an absence of publication bias in the results concerning community-based interventions for older adults living alone (Table 3).

## DISCUSSION

In this study, we conducted a systematic review and analysis of the effectiveness of community-based interventions for older adults who reside alone. We first identified relevant studies from 3 databases, with 9 studies ultimately included for review. Following an assessment of risk of bias, we performed a meta-analysis to determine the effect sizes associated with the community-based interventions. To facilitate comparison of the various types of interventions and their outcomes, we organized both interventions and outcomes into 5 distinct categories and evaluated their effect sizes individually. The overall risk of bias was reported to be fairly low, which can be attributed to the inclusion of 5 non-RCTs along with RCTs that lacked assessor blinding. To mitigate this bias, each non-RCT was appraised using a standardized assessment tool. Consequently, although a risk of bias was detected, it was relatively small, since the 9 studies implemented a total of 11 interventions.

Combined intervention was defined as involving 2 or more of the following categories: physical activity, nutrition, social relationships, and social participation. This combined type was the most frequently applied among participants, followed by physical activity and social relationship interventions. It also ranked as the second most effective, having been implemented in 5 studies and yielding 16 measured outcomes. In contrast, interventions focusing solely on nutrition and social participation were rare. Notably, when nutrition intervention was applied independently, it demonstrated the largest effect size, which was statistically significant. However, given that only a single study employed this intervention, one should interpret these results with caution before concluding that it is the most beneficial for older adults living alone. While other intervention types exhibited relatively low effect sizes when evaluated individually, their effectiveness was markedly enhanced when combined. For instance, the social participation intervention had the smallest effect size, which did not reach statistical significance. However, it was usually included among combined interventions. Overall, it appears that combined approaches may be particularly effective for older adults living alone.

Regarding the effect sizes of outcome measurements, health behavior outcomes were the most impactful among the participants. These included self-care behavior, dietary habits, nutritional knowledge, and nutrition status. Six health behavior outcomes were measured following the implementation of combined interventions. Apart from health behavior, the outcomes related to mental health (such as loneliness, cognition, depression, and feelings of hopelessness) and perceived health (including self-efficacy, fall efficacy, perceived stress, and coping strategies) demonstrated medium effect sizes. These effect sizes were calculated based on 5 mental health outcomes and 4 perceived health outcomes, which were evaluated by 5 studies and 3 studies, respectively, for each type of intervention. Since 4 outcomes of the 5 outcomes were measured following combined interventions, such interventions appear to be useful in improving the health behavior, mental health, and perceived health of older adults who live alone.

Previous research indicated that community-based interventions are effective in promoting health, yet no statistical evidence was provided to support this conclusion [25]. In contrast, in the present study, we analyzed health-promoting interventions involving various lifestyle factors and determined the effect size of each subgroup. To facilitate successful aging, healthy lifestyle interventions are imperative, particularly among older adults who live alone. Consequently, it is essential to provide not only financial assistance but also support for health promotion to prevent these individuals from feeling isolated and to increase their motivation to participate in daily activities and occupations [1]. For community-based interventions, a combined approach that includes physical activity, nutrition, social relationships, and social participation is considered the most beneficial for this demographic. Moreover, such an integrated intervention is likely to be the most effective in improving various aspects of health behavior and mental health, encompassing physical, social-emotional, and perceived health.

The findings of this study reveal that community-based interventions had a substantial impact on health behavior, with a large effect size, along with a moderate effect size on mental health and perceived health. These results suggest that the interventions were successful in promoting healthy lifestyles among older adults residing alone within their communities. Additionally, the data imply that combined interventions are the most beneficial for this demographic. Consequently, our results could serve as a foundation for the development of future policies designed to promote healthy living among older adults living independently, while also mitigating potential risk factors.

This study presented 3 noteworthy limitations. First, the review was restricted to studies published in English, despite the fact that numerous Asian and European countries are likewise grappling with aging populations. Second, the inclusion of non-RCTs introduced the potential for risk bias. Finally, the number of studies examined was relatively small. Consequently, future research should include a sufficient number of studies pertaining to the 5 distinct intervention types, incorporating works in various languages and those involving RCTs.

## CONCLUSION

In this study, we performed a meta-analysis to measure the effectiveness of community-based interventions and their outcomes among older adults who live alone. Additionally, we classified various interventions and suggested a direction for future community-based approaches. The findings indicated that combined intervention was the most effective, as it had the strongest effects on improving the mental health of the participants. Consequently, this study proposes the implementation of combined community-based interventions for older adults living alone, to improve their health behavior, their mental health, and potentially their physical and social-emotional well-being.

## Figures and Tables

**Table 1.** Statistical heterogeneity test for community-based interventions for older adults living alone

Variables	No. of data	Overall effect size	Statistical heterogeneity test			
			Q	df(Q)	p-value	I^2^
Physical activity	10	0.33 (0.20, 0.46)*	9.63	9	0.00	6.51
Nutrition	5	0.96 (0.67, 1.25)*	7.03	4	0.00	43.12
Social relationship	5	0.29 (0.06, 0.52)*	1.37	4	0.02	0.00
Social participation	2	0.20 (-0.09, 0.48)	0.05	1	0.17	0.00
Combined	16	0.43 (0.26, 0.60)*	64.43	15	0.00	76.72
Overall	38	0.38 (0.32, 0.54)*	115.27	37	0.00	67.90

CI, confidence interrval; df, degrees of freedom.

* p<0.05.

**Table 2.** Statistical heterogeneity test for the outcomes of the community-based interventions for older adults

Variables	No. of data	Overall effect size	Statistical heterogeneity test			
			Q	df(Q)	p-value	I^2^
Health behavior	6	0.98 (0.73, 1.22)*	7.25	5	0.00	31.09
Mental health	5	0.68 (0.19, 1.16)*	22.20	4	0.01	81.98
Perceived health	4	0.48 (-0.03, 0.98)	14.02	3	0.07	78.60
Physical health	10	0.32 (0.20, 0.44)*	7.25	9	0.00	0.00
Social-emotional health	13	0.24 (0.13, 0.35)*	18.28	12	0.00	34.35
Overall	38	0.43 (0.32, 0.54)*	115.27	37	0.00	67.90

CI, confidence interrval; df, degrees of freedom.

* p<0.05.

**Table 3.** Trim and fill test to assess publication bias

Variables	Random effect		
	Point estimate	Lower estimate	Upper estimate
Right side			
Observed values	0.43	0.32	0.54
Adjusted values	0.47	0.36	0.59
Left side			
Observed values	0.43	0.32	0.54
Adjusted values	0.43	0.32	0.54

## Supplementary materials

Supplementary Material 1. General characteristics of community-based intervention studies

## References

[1] Rolls L, Seymour JE, Froggatt KA, Hanratty B (2011). Older people living alone at the end of life in the U.K.: research and policy challenges. Palliat Med.

[2] https://www.census.gov/newsroom/press-releases/2022/americas-families-and-living-arrangements.html.

[3] https://www.msdmanuals.com/professional/geriatrics/social-issues-in-older-adults/effects-of-lifetransitions-on-older-adults.

[4] Ko H, Park YH, Cho B, Lim KC, Chang SJ, Yi YM (2019). Gender differences in health status, quality of life, and community service needs of older adults living alone. Arch Gerontol Geriatr.

[5] Lee J, Ham MJ, Pyeon JY, Oh E, Jeong SH, Sohn EH (2017). Factors affecting cognitive impairment and depression in the elderly who live alone: cases in Daejeon Metropolitan City. Dement Neurocogn Disord.

[6] Lim EJ (2013). The relationship between depression, cognitive function and the instrumental activities of daily living of elderly women living alone. J Korea Acad Ind Coop Soc.

[7] Bergland AM, Tveit B, Gonzalez MT (2016). Experiences of older men living alone: a qualitative study. Issues Ment Health Nurs.

[8] Hung YC, Chen YH, Lee MC, Yeh CJ (2021). Effect of spousal loss on depression in older adults: impacts of time passing, living arrangement, and spouse’s health status before death. Int J Environ Res Public Health.

[9] Lou VW, Ng JW (2012). Chinese older adults’ resilience to the loneliness of living alone: a qualitative study. Aging Ment Health.

[10] Messinger-Rapport BJ, Thacker HL (2001). Prevention for the older woman. A practical guide to assessing physical and cognitive function. Geriatrics.

[11] Das Gupta D, Kelekar U, Rice D (2020). Associations between living alone, depression, and falls among community-dwelling older adults in the US. Prev Med Rep.

[12] Kharicha K, Iliffe S, Harari D, Swift C, Gillmann G, Stuck AE (2007). Health risk appraisal in older people 1: are older people living alone an “at-risk” group?. Br J Gen Pract.

[13] Lage I, Braga F, Almendra M, Meneses F, Teixeira L, Araujo O (2022). Falls in older persons living alone: the role of individual, social and environmental factors. Enferm Clin (Engl Ed).

[14] Hwang MJ (2016). Determinant of poverty among the elderly living alone: using decision tree analysis. J Knowl Inf Technol Syst.

[15] Kramarow EA (1995). The elderly who live alone in the United States: historical perspectives on household change. Demography.

[16] Mui AC, Burnette JD (1994). A comparative profile of frail elderly persons living alone and those living with others. J Gerontol Soc Work.

[17] https://www.oecd-ilibrary.org/sites/fb958d50-en/index.html?itemId=/content/component/fb958d50-en.

[18] https://www.oecd-ilibrary.org/sites/d76e4fad-en/index.html?itemId=/content/component/d76e4fad-en.

[19] Hanley A, Silke C, Murphy J (2011). Community-based health efforts for the prevention of falls in the elderly. Clin Interv Aging.

[20] Li X, Wang B, Tan D, Li M, Zhang D, Tang C (2018). Effectiveness of comprehensive social support interventions among elderly patients with tuberculosis in communities in China: a communitybased trial. J Epidemiol Community Health.

[21] Baldwin C, Dendle K, McKinlay A (2019). Initiating senior co-housing: people, place, and long-term security. J Hous Elderly.

[22] https://www.housinglin.org.uk/_assets/Resources/Housing/OtherOrganisation/senior-cohousing-communities-full.pdf.

[24] Price D (2006). The poverty of older people in the UK. J Soc Work Pract.

[25] Ilgaz A, Gözüm S (2019). Health promotion interventions for older people living alone: a systematic review. Perspect Public Health.

[26] Elliott DS, Millstein SG, Petersen AC, Nightingale EO (1993). Promoting the health of adolescents: new directions for the twenty-first century.

[27] Hwang JE (2010). Reliability and validity of the health enhancement lifestyle profile (HELP). OTJR (Thorofare N J).

[28] Park JH, Park KH, Han DS (2021). The Yonsei Lifestyle Profile for adults and the older adults: development and test‐retest reliability. Alzheimers Dement.

[29] Byeon H (2019). Relationship between physical activity level and depression of elderly people living alone. Int J Environ Res Public Health.

[30] Kim HS (2017). Effect of pain, nutritional risk, loneliness, perceived health status on health-related quality of life in elderly women living alone. J Korea Converg Soc.

[31] Payette H, Coulombe C, Boutier V, Gray-Donald K (2000). Nutrition risk factors for institutionalization in a free-living functionally dependent elderly population. J Clin Epidemiol.

[32] Burnette D, Mui AC (1994). Determinants of self-reported depressive symptoms by frail elderly persons living alone. J Gerontol Soc Work.

[33] Joo CL, Park JJ, Kim A, Park NL, Lim J, Park HS (2019). Health behaviors and lifestyle patterns of elderly living alone in Korea. Korean J Fam Pract.

[34] Tong H, Lai DW, Walsh CA (2019). Formal social participation and utilization of community-based services among urban elderly Chinese living alone in Shanghai, China. J Soc Serv Res.

[35] Hedges LV (1992). Meta-analysis. J Educ Stat.

[36] Page MJ, McKenzie JE, Bossuyt PM, Boutron I, Hoffmann TC, Mulrow CD (2021). The PRISMA 2020 statement: an updated guideline for reporting systematic reviews. Int J Surg.

[37] Ahn JA, Park J, Kim CJ (2018). Effects of an individualised nutritional education and support programme on dietary habits, nutritional knowledge and nutritional status of older adults living alone. J Clin Nurs.

[38] Downs SH, Black N (1998). The feasibility of creating a checklist for the assessment of the methodological quality both of randomised and non-randomised studies of health care interventions. J Epidemiol Community Health.

[39] Bowden J, Tierney JF, Copas AJ, Burdett S (2011). Quantifying, displaying and accounting for heterogeneity in the meta-analysis of RCTs using standard and generalised Q statistics. BMC Med Res Methodol.

[40] Higgins JP, Thomas J, Chandler J (2019). Cochrane handbook for systematic reviews of interventions.

[41] Aydın M, Kutlu FY (2021). The effect of group art therapy on loneliness and hopelessness levels of older adults living alone: a randomized controlled study. Florence Nightingale J Nurs.

[42] Cederbom S, Leveille SG, Bergland A (2019). Effects of a behavioral medicine intervention on pain, health, and behavior among community-dwelling older adults: a randomized controlled trial. Clin Interv Aging.

[43] Ristolainen H, Kannasoja S, Tiilikainen E, Hakala M, Närhi K, Rissanen S (2020). Effects of ‘participatory group-based care management’ on wellbeing of older people living alone: a randomized controlled trial. Arch Gerontol Geriatr.

[44] Zingmark M, Fisher AG, Rocklöv J, Nilsson I (2014). Occupation-focused interventions for well older people: an exploratory randomized controlled trial. Scand J Occup Ther.

[45] Jung H, Lee JE (2017). The impact of community-based eHealth self-management intervention among elderly living alone with hypertension. J Telemed Telecare.

[46] Kil T, Yoon KA, Ryu H, Kim M (2019). Effect of group integrated intervention program combined animal-assisted therapy and integrated elderly play therapy on live alone elderly. J Anim Sci Technol.

[47] Lorente-Martínez R, Brotons-Rodes P, Sitges-Maciá E (2022). Benefits of a psychosocial intervention programme using volunteers for the prevention of loneliness among older women living alone in Spain. Health Soc Care Community.

[48] Song MS, Boo S (2022). Effects of a nurse-led multicomponent intervention for frail older adults living alone in a community: a quasi-experimental study. BMC Nurs.

