# Supplementary material for: Effectiveness of community-based interventions for older adults living alone: a systematic review and meta-analysis
Source: Epidemiol Health. 2024 Jan 3;46:e2024013. doi: 10.4178/epih.e2024013 (PMC11040216; doi:10.4178/epih.e2024013)
Supplement: Supplementary Material 1. — General characteristics of community-based intervention studies [file epih-46-e2024013-Supplementary-1.docx]

**Supplementary material 1.** General characteristics of community-based intervention studies

| Author (Year) | Participants | | | | Intervention | | Outcomes | | Downs and Black checklist score |
| --- | --- | --- | --- | --- | --- | --- | --- | --- | --- |
|  | EG | | CG | | Intervention type | Intervention | Outcome type | Assessment tool |  |
| Ristolainen, et al.(2020) | N = 185  Mean age = 76.8 (SD 7.2)  Woman N(%) = 152 (82.2) | | N = 207  Mean age = 76.8 (SD 7.76)  Woman N(%) = 173 (83.6) | | Combined | Group-based care management activity   - Social support - Counseling - Education in health behavior | Social-emotional health | WHO Quality of Life-BREF (WHOQOL-Brief) | 32 |
|  |  |  |  |  |  |  | Mental health | 12-item form of the Revised UCLA Loneliness Scale |  |
|  |  |  |  |  |  |  | Social-emotional health | generalized trust-trust in other people |  |
|  |  |  |  |  |  |  | Social-emotional health | generalized trust-mistrust other people |  |
|  |  |  |  |  |  |  | Social-emotional health | institutional trust |  |
| Lorente-Martínez, et al.(2022) | N = 34  Mean age = 77.62  (SD 7.86) | | N = 14  Mean age = 77.36 (SD 7.91) | | Social relationship | Psychosocial intervention program   - Psychosocial support - Social connection | Perceived health | Self-efficacy in Ageing Scale | 27 |
|  |  |  |  |  |  |  | Social-emotional health | Subjective Social Participation Index (SSPI) |  |
|  |  |  |  |  |  |  | Mental health | UCLA Loneliness Scale (Version 3) |  |
| Kil, et al.(2019) | N = 10  Age: 70-81  Woman N(%) = 6 (60%) | | N = 10  Age: 70-81  Gender (female, %) = 5 (50%) | | Combined | Animal-Assisted Therapy (AAT) and Elderly Play Therapy (IEPT)   - Social interaction activities - Cognitive activities - Emotional activities - Physical activities | Mental health | Trail making test-A  (TMT-A) | 27 |
| Jung, et al.(2017) | N = 31  Age: 80.9 (SD 6.6)  Woman N(%) = 24 (77.4) | | N = 33  Age: 81.2 (SD 4.1)  Woman N(%) = 29 (87.9%) | | Combined | Community-based eHealth monitoring and monthly telephone counseling   - Self-care behavior (healthy diet, exercise, smoking cessation, medication, and emotional support). - Manage hypertension (overview of hypertension, healthy lifestyle, medication) | Perceived health | Self-efficacy(11-item scale to measure) | 27 |
|  |  |  |  |  |  |  | Health behavior | Self-care behaviors (10-item self-reported developed by Sung and Kim) |  |
|  |  |  |  |  |  |  | Social-emotional health | Multidimensional Scale of Perceived Social Support |  |
| Aydın, et al.(2021) | N = 60  Age: 72.56 (SD 1.01)  Woman N(%) = 47 (78.3) | | N = 30  Age: 72.6 (SD 8.1)  Woman N(%) = 23 (76.7) | | Combined | Group art therapy using clay   - Emotional support, clay activity, | Mental health | The UCLA-Loneliness Scale (UCLA-LS) | 28 |
| Ahn, et al.(2018) | N = 37 | | N = 34 | | Nutrition | Nutritional Education and Support program (NESP)   - Dietary habits - Nutritional knowledge - Nutritional intake status | Health behavior | Protein intake (Computer Aided Nutritional Analysis Program 5.0(CAN Pro, Korea Nutrition Society) | 26 |
|  |  |  |  |  |  |  | Health behavior | Calcium intake (Computer Aided Nutritional Analysis Program 5.0 (CAN Pro, Korea Nutrition Society) |  |
|  | Age: 77.61 (SD 5.38)  Woman N(%) = 58 (81.7) | | | |  |  | Health behavior | Vitamin A (Computer Aided Nutritional Analysis Program 5.0 (CAN Pro, Korea Nutrition Society) |  |
|  |  |  |  |  |  |  | Health behavior | Vitamin B2 (Computer Aided Nutritional Analysis Program 5.0(CAN Pro, Korea Nutrition Society) |  |
|  |  |  |  |  |  |  | Health behavior | Vitamin C (Computer Aided Nutritional Analysis Program 5.0 (CAN Pro, Korea Nutrition Society) |  |
| Zingmark, et al.(2014) | N = 46  Age (range) 79 = (77-82)  Woman (%)= 82.6 | N = 41  Age (range) = 77-82)  Woman (%) = 82.9 | N = 49  Age (range) = 79 (77-82)  Woman (%) = 81.6 | N = 41  Age (range) = 79 (77-82)  Woman (%) = 82.9 | Physical activity | Occupation-focused individual interventions (Client-centred collaboration)   - Maintaining meaningful activities | Social-emotional health | Modified NPS interest checklist (MNPS)(leisure engagement) | 29 |
|  |  |  |  |  |  |  | Physical health | ADL Taxonomy (ADL ability) |  |
|  |  |  |  |  | Social participation | Occupation-focused activity interventions (Engagement in occupation)   - Engaging activities | Social-emotional health | Modified NPS interest checklist (MNPS)(leisure engagement) |  |
|  |  |  |  |  |  |  | Physical health | ADL Taxonomy (ADL ability) |  |
|  |  |  |  |  | Social relationship | Occupation-focused activity interventions (Education)   - Learning and discussing about healthy aging and health promotion | Social-emotional health | Modified NPS interest checklist (MNPS)(leisure engagement) |  |
|  |  |  |  |  |  |  | Physical health | ADL Taxonomy (ADL ability) |  |
| Song, et al.(2022) | N = 62  Age = 79.56 (SD 5.5)  Woman N(%) = 56 (90.3) | | N = 64  Age = 78.05 (SD 5.21)  Woman N(%) = 58 (90.6) | | Combined | Multicomponent intervention   - exercise, cognitive training, and education for nutrition and management frailty | Physical health | 28-item frailty index (Frailty) | 27 |
|  |  |  |  |  |  |  | Physical health | Timed up & go(sec) |  |
|  |  |  |  |  |  |  | Physical health | Handgrip strength(kg) |  |
|  |  |  |  |  |  |  | Mental health | Geriatric Depression Scale-Short Form Korean Version (GDSSF-K) (depression) |  |
|  |  |  |  |  |  |  | Social-emotional health | Social activities (A five-item social activity scale) |  |
|  |  |  |  |  |  |  | Social-emotional health | Medical Outcomes Study Social Support Scale (Social support) |  |
| Cederbom, et al.(2019) | N = 52  Age = 85.2 (SD 5.6)  Woman (%) = 87.6 | | N = 53  Age = 85.4 (SD 6.7)  Woman (%) = 93 | | Physical activity | Behavioral medicine intervention (Physical Therapy (PT))   - Physical activity | Physical health | Brief Pain Inventory short version, Norwegian version (BPI) | 30 |
|  |  |  |  |  |  |  | Physical health | Norwegian version of the Short Physical Performance Battery (SPPB) |  |
|  |  |  |  |  |  |  | Physical health | pain severity items in the BPI and consists of four items |  |
|  |  |  |  |  |  |  | Perceived health | Coping Strategies Questionnaire, Norwegian version (Catastrophizing thoughts, CAT 2 item) |  |
|  |  |  |  |  |  |  | Perceived health | Falls-Efficacy Scale International (FES-I) version |  |
|  |  |  |  |  |  |  | Physical health | Physical activity level, GrimbyFrändin scale (physical activity including household activities) |  |
|  |  |  |  |  |  |  | Social-emotional health | PCS, Norwegian version of the 12-item Short-Form Health Survey, SF-12 |  |
|  |  |  |  |  |  |  | Social-emotional health | MCS, Norwegian version of the 12-item Short-Form Health Survey, SF-12 |  |

EG, Experimental Group; CG, Control Group; N, Number of sample; SD, Standard Deviation
